# Statin use is associated with higher white matter hyperintensity volumes and lower grey matter volumes

**DOI:** 10.1093/braincomms/fcae417

**Published:** 2024-11-20

**Authors:** Mélissa Gentreau, Maud Miguet, Oreste Affatato, Gull Rukh, Helgi Birgir Schiöth

**Affiliations:** Functional Pharmacology and Neuroscience, Department of Surgical Sciences, University of Uppsala, 751 23 Uppsala, Sweden; Functional Pharmacology and Neuroscience, Department of Surgical Sciences, University of Uppsala, 751 23 Uppsala, Sweden; Sport, Physical Activity, Rehabilitation and Movement for Performance and Health (SAPRéM), Université d’Orléans, 45067 Orléans, France; Functional Pharmacology and Neuroscience, Department of Surgical Sciences, University of Uppsala, 751 23 Uppsala, Sweden; Functional Pharmacology and Neuroscience, Department of Surgical Sciences, University of Uppsala, 751 23 Uppsala, Sweden; Functional Pharmacology and Neuroscience, Department of Surgical Sciences, University of Uppsala, 751 23 Uppsala, Sweden

**Keywords:** cholesterol, hydroxymethylglutaryl-CoA reductase inhibitors, structural magnetic resonance imaging, temporal lobe, white matter lesions

## Abstract

While statins are routinely prescribed to prevent cardiovascular diseases, their effects on brain alterations remain largely unknown. Very few studies have examined the differences in brain volumes between statin users and non-users, and existing research has yielded inconsistent results. This cross-sectional study aims to investigate the association between statin use at baseline and global and specific brain volumes measured 9 years later in a large population-based sample of middle-aged and older adults. Participants from the UK Biobank without neurological and psychiatric disorders consisted of 3285 statin users (mean 60 years and 69% males) and 36 229 non-users (mean 55 years and 46% males). We used linear models to estimate the mean volumetric differences between statin users and non-users while adjusting for UK Biobank assessment centre, age, sex, ethnicity, education, apolipoprotein E ɛ4 status, Townsend deprivation index, antidepressant use, intracranial volume, lifestyle factors (alcohol intake frequency, smoking and physical activity) and health-related conditions (body mass index, blood pressure, diabetes, coronary heart disease, stroke, head injury, depression and insomnia). Moreover, mediation analysis was performed to evaluate whether the association between statin use and global brain volumes was mediated by total serum cholesterol concentration. Statin use was associated with lower grey matter volume [β = −1575 mm^3^ (−2358, −791)], with 20% of this association mediated by total serum cholesterol concentration. Statin use was also associated with lower peripheral cortical grey matter volumes [β = −1448 mm^3^ (−2227, −668)] and higher white matter hyperintensity [β = 0.11 mm^3^ (0.07, 0.15)]. However, white matter volume did not differ significantly between statin users and non-users. Further analyses revealed that volumes of thalamus, pallidum, hippocampus, nucleus accumbens and other regions of the temporal lobe were smaller among statin users compared with non-users. This study showed that statin use is associated with higher white matter hyperintensity volumes and lower total and peripheral cortical grey matter volumes 9 years later, indicative of the brain’s ageing process. Moreover, the observed grey matter alterations were partially explained by statin-induced total serum cholesterol reduction. This study emphasizes the potential direct and indirect effects of statins on brain volume.

## Teaser

Gentreau *et al*. report that statin use is associated with higher white matter hyperintensity volumes and lower total and peripheral cortical grey matter volumes measured 9 years later. The decrease in grey matter volume by statin use was partially mediated by total serum cholesterol reduction.

## Graphical Abstract

**Graphical Abstract:**
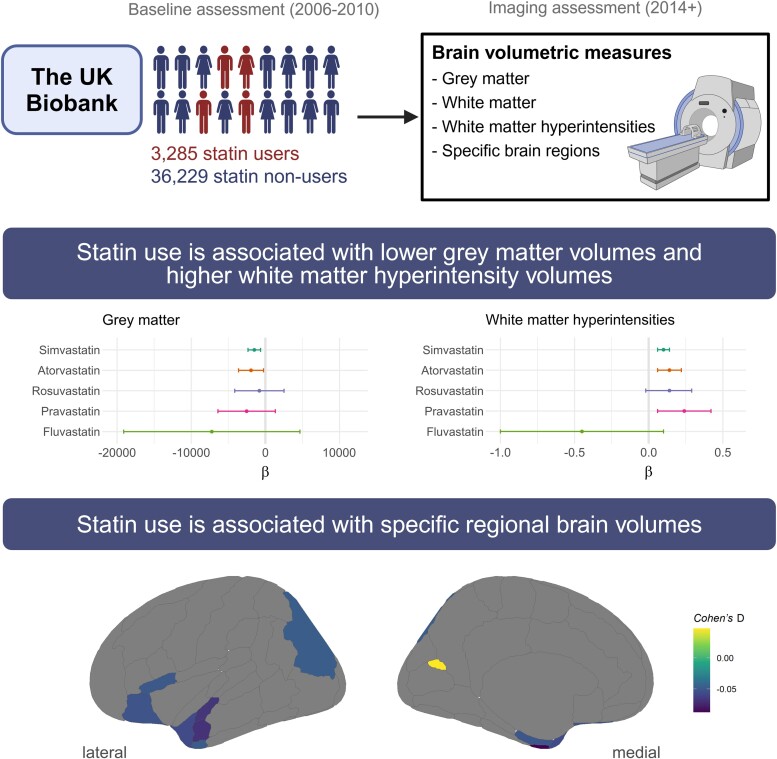

## Introduction

Brain structure alterations are indicative of the ageing process,^1^ which sometimes involves pathological processes that accelerate brain ageing. One such indicator is the white matter hyperintensity (WMH), resulting from chronic ischaemia associated with cerebral small vessel disease.^2^ The prevention and treatment of brain ageing are needed to enhance the proportion of healthy living and reduce the risks of future strokes, cognitive decline and neurodegenerative diseases among the ageing population.^3^ Brain ageing is multifactorial, and observational studies highlighted that cardiovascular health status is an important determinant.^4-6^ In this context, lipid-lowering agents, such as statins, have been widely and routinely prescribed as an effective medication for reducing plasma low-density lipoprotein cholesterol in primary and secondary prevention of cardiovascular diseases.^7^

Cholesterol is a major constituent of myelin and neuronal membranes. Hence, it plays a fundamental role in various brain processes, including signal transmission, synaptic function and lipid raft formation.^8,9^ It is usually postulated that cholesterol biosynthesis in the brain operates independently of peripheral cholesterol metabolism,^9^ but some studies suggest that statin might modify brain cholesterol metabolism.^10,11^ In the brain, cholesterol is primarily synthesized by astrocytes and transported to neurons through binding to apolipoprotein E.^12^ The presence of the apolipoprotein E ɛ4 allele (*APOE4*) has been shown to increase plasma low-density lipoprotein concentration and the risk of atherosclerosis.^13^ In addition, *APOE4* is the main genetic risk factor for Alzheimer’s disease, contributing to reduced brain cholesterol transport and the accelerated onset of senile plaques.^12,14^ Recently, *APOE4* status has been associated with lower grey matter (GM) volume, especially in the hippocampus and the entorhinal cortex.^15^ Thus, *APOE4* status must be considered in studies evaluating the association between statin use and brain structure alterations.

Overall, the effects of statin use on brain structure remain largely unknown. Most studies have primarily investigated the association between statin use and WMH, yielding conflicting results. Although a cohort study with 4 years of follow-up observed a larger increase in WMH among statin users,^16^ most studies reported either no^17-20^ or negative association between statin use and WMH volumes.^21,22^ Beyond WMH, only one study has evaluated the association of statin use with global brain volumes, such as GM and white matter (WM) volumes,^20^ and two studies examined specific brain regions like the hippocampus,^18,23^ a hallmark of brain ageing and known to atrophy in Alzheimer’s disease.^24^ Overall, these studies have been conducted on specific populations (e.g. older adults, hypertensive patients and participants with cardiovascular risk factors) and their sample sizes have been relatively small (ranging from 33 to 732), limiting our ability to draw firm conclusions. Therefore, large-scale epidemiological studies are needed to examine the association between statin use and volumes of GM, WM, WMH and specific brain regions in a healthier and younger population.

Previously, our research showed that statin use was cross-sectionally associated with lower cognitive performance and this association was mediated by plasma low-density lipoprotein cholesterol concentration.^25^ Thus, the present cross-sectional study aimed (i) to evaluate the association between statin use and volumes of GM, WM, peripheral cortical GM and WMH in a large population-based sample of middle-aged and older adults; (ii) to determine whether total serum cholesterol concentration mediated these associations; and (iii) to further investigate the association between statin use and specific cortical and subcortical brain regions.

## Materials and methods

### Study sample

**Figure 1:**
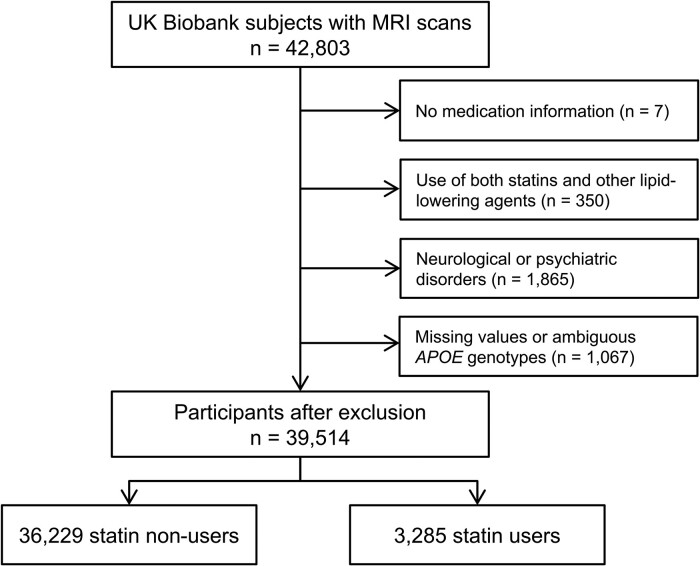
Flow chart for selection of the study participants.

The UK Biobank is a large population-based prospective cohort. At baseline (between 2006 and 2010), approximately half a million middle-aged and older participants across the UK were recruited. A wide variety of information from lifestyle phenotypes to genetic and biological data was gathered.^26^ In 2014, the UK Biobank imaging study began and is still ongoing.^27^ The ethics approval of the UK Biobank project was granted by the North West Multi-centre Research Ethics Committee, and the use of the data was further approved by the Regional Ethics Committee of Uppsala, Sweden. All participants provided written consent with the right to withdraw at any time. The present study used data from participants with brain MRI, released in January 2023 (*n* = 42 803). We excluded all the participants lacking medication information (*n* = 7), using both statins and other lipid-lowering medications (*n* = 350), having prevalent neurological or psychiatric disorders (*n* = 1865) and having missing values or ambiguous *APOE* genotypes (*n* = 1067). After the selection process, the final sample comprised 3285 statin users and 36 229 non-users at baseline (Fig. 1).

### Medication use

Current prescription medications (weekly, monthly or three monthly) were reported at baseline and the imaging visit. The type and number of medications taken were collected using a touchscreen questionnaire, verified by a trained nurse during a verbal interview and reported as medication codes. Medications identified as statin were recoded in a single binary variable (statin use: yes/no) as previously described.^25^ Similarly, antidepressant medications were grouped in one binary variable (yes/no).

### Brain MRI

Identical 3 T Siemens Skyra scanners, dedicated to the UK Biobank project, were used to perform MRI examinations at four centres (Cheadle, Reading, Newcastle and Bristol). The present study made use of imaging-derived phenotypes generated by an image-processing pipeline developed and run on behalf of the UK Biobank.^28^ Specifically, we used total GM, total WM and peripheral cortical GM volumes from T_1_-weighted imaging (voxel dimensions = 1 1 × 1 mm^3^; matrix size = 208 × 256 × 256; three-dimensional MPRAGE, sagittal, *R* = 2, TI/TR = 880/2000 ms) and WMH from T_2_-weighted fluid-attenuated inversion recovery (FLAIR) imaging (voxel dimensions = 1.05 × 1.0 × 1.0 mm^3^; matrix size = 192 × 256 × 256, FLAIR, 3D SPACE, sagittal, *R* = 2, PF 7/8, fat sat, TI/TR = 1800/500 ms, elliptical), which reflects demyelination and axon loss.^2^ Additionally, we used 48 cortical volumes and 7 subcortical volumes generated from FAST and FIRST segmentation, respectively.^29^ All volumes are expressed in mm^3^.

### Confounding variables

Information related to baseline characteristics, such as age, sex, socioeconomic status and education level, was collected at the time of initial assessment. Townsend deprivation index (TDI) was used as a proxy of socioeconomic status. Ethnicity was defined as self-reported White background versus non-White (i.e. Mixed, Asian, Black, Chinese and other ethnic groups). Education level was recoded into a dichotomous variable, defined as having a college or university degree versus the others as previously described.^30^

Information related to lifestyle factors was extracted from a touchscreen questionnaire. Alcohol intake frequency was categorized into six groups (daily or almost daily, three or four times a week, once or twice a week, one to three times a month, special occasions only and never). Smoking status was classified as never, past or current. Physical activity was defined as number of days per week of moderate activity and was recoded into categories of low (0–1), medium (2–4) and high (5–7).

Health-related conditions, including weight, height, blood pressure and history of medical conditions, were also recorded during the baseline assessment. Body mass index (BMI) was calculated as weight/height² (kg/m²). Systolic blood pressure (SBP) and diastolic blood pressure (DBP) were measured using Omron HEM-705 IT digital blood pressure monitor, twice with 1-min interval between the two measurements. The average SBP and DBP were calculated and used in the analyses. Serum cholesterol concentration was quantified using routine enzymatic methods.^31^ History of diabetes, stroke, coronary heart disease (CHD), head injury and depression were self-reported and recoded into binary variables (yes/no). Sleeplessness/insomnia was reported as ‘never/rarely’, ‘sometimes’ or ‘usually’.

*APOE* genotypes were determined based on the two single nucleotide polymorphisms (SNPs), rs429358 and rs7412 as described previously.^25^ Participants with ambiguous genotypes (ɛ2/ε4 or ɛ1/ɛ3) were excluded. *APOE4* status was defined as carrying at least one ɛ4 allele.

### Statistical analysis

#### Main analysis

First, we evaluated the associations between statin use and global brain volumes (GM, WM, peripheral cortical GM and WMH volumes as separate dependent variables) using linear regression models. WMH was log-transformed to fit a normal distribution. Models were adjusted for various covariates based on the literature, to reduce the bias due to confounding. We estimated three nested models, sequentially adjusting for all covariates to account for the modification in the variance explained (adjusted *R*²) and the robustness of the associations. Model I (basic model) considers sociodemographic characteristics, genetic risk factors and factors affecting brain volumes.^32^ It included UK Biobank imaging centre, age, sex, ethnicity, education, TDI, *APOE4* status, antidepressant use and intracranial volume (total brain volume + cerebrospinal fluid volume). Model II included all covariates in Model I and lifestyle factors (alcohol intake frequency, smoking status and physical activity). Model III included all covariates in Model II and health-related conditions (BMI, SBP, DBP, diabetes, CHD, stroke, head injury, depression and insomnia). Missing values for confounding factors (ranging from 0 to 3.2%) were excluded from the analyses. In addition, Model III was replicated according to statin type (simvastatin, atorvastatin, rosuvastatin, pravastatin and fluvastatin).

#### Mediation analysis

Second, we conducted a mediation analysis to evaluate whether total serum cholesterol concentration mediated the association between statin use and global brain volumes as described elsewhere.^25^ We used Model III covariates. The average direct effect, indirect effect, total effect, proportion mediated and their confidence intervals were computed using mediation package in R with 1000 simulations.^33^

#### Secondary analysis

Furthermore, we evaluated the association between statin use and the volumes of the cortical and the subcortical brain regions using linear regression models. *P*-values were corrected for multiple testing with false discovery rate correction.^34^ To facilitate the comparison of association across the different brain regions, we standardized the β coefficients according to Cohen’s definition.^35^ Spatial representation of the statistics was plotted using *ggseg* package in R.^36^

#### Sensitivity analysis

A first sensitivity analysis was conducted after excluding participants who initially reported statin use at recruitment but indicated no statin use during the imaging visit (*n* = 828), as well as those who initiated statin use at the imaging visit (*n* = 4817). Hence, we specifically assessed the association in participants who consistently used from recruitment to the imaging visit (considered as long-term statin users) versus those who never used statins.

A second sensitivity analysis was conducted to address potential recall bias in self-reported health-related conditions (diabetes, CHD, stroke, head injury, depression and insomnia). Diagnoses based on the International Classification of Diseases—10th revision (ICD-10) were used to identify prevalent cases of these conditions. Self-reported variables were replaced with variables based on ICD-10 diagnoses in Model III of the main analysis.

All statistical analyses were carried out with R version 4.2.1 (The R Foundation for Statistical Computing, Vienna, Austria; www.R-project.org).

## Results

### Baseline characteristics

The cross-sectional analysis included 3285 statin users and 36 229 non-users. The median follow-up time between baseline and the imaging assessment was 9.2 (interquartile range = 2.7) years. The participants’ mean age was 55.0 (SD = 7.6), and 47.5% were male. Compared with non-users, statin users were older and more likely to be male. They were less likely to have a college or university degree and more likely to carry *APOE4*. They tended to have a higher BMI and blood pressure. Those who reported history of diabetes, stroke, CHD or hypertension were more frequent among statin users (Table 1).

**Table 1:** Characteristics of the study sample at baseline

Characteristics	Total sample	Statin non-users	Statin users
Mean (SD) or *n* (%)	*n* = 39 514	*n* = 36 229	*n* = 3285
Age	55.0 (7.6)	54.5 (7.5)	60.2 (6.0)
Sex (men)	18 763 (47.5)	16 484 (45.5)	2279 (69.4)
College or university degree	18 519 (46.9)	17 240 (47.6)	1279 (38.9)
TDI^a^	−2.61 (3.41)	−2.61 (3.42)	−2.61 (3.29)
Ethnic background			
White	38 238 (96.8)	35 082 (96.8)	3156 (96.1)
Mixed	174 (0.4)	162 (0.5)	12 (0.4)
Asian or Asian British	405 (1.0)	335 (0.9)	70 (2.1)
Black and Black British	250 (0.6)	239 (0.7)	11 (0.3)
Chinese	117 (0.3)	115 (0.3)	2 (0.1)
Other ethnic group	203 (0.5)	182 (0.5)	21 (0.6)
*APOE4* status	9995 (25.3)	9073 (25.1)	922 (28.1)
Antidepressant use	2267 (5.7)	2022 (5.6)	245 (7.5)
Alcohol use			
Almost daily	9016 (22.8)	8131 (22.4)	885 (26.9)
3–4 times/week	11 127 (28.2)	10 242 (28.3)	885 (26.9)
1–2 times/week	10 119 (25.6)	9359 (25.8)	760 (23.1)
1–3 times/month	4281 (10.8)	3958 (10.9)	323 (9.8)
On special occasions	3172 (8.0)	2915 (8.1)	257 (7.8)
Never	1785 (4.5)	1610 (4.4)	175 (5.3)
Smoking status			
Never	24 106 (61.0)	22 448 (62.0)	1658 (50.5)
Previous	12 910 (32.7)	11 521 (31.8)	1389 (42.3)
Current	2419 (6.1)	2190 (6.0)	229 (7.0)
Physical activity (days/week)			
0–1	8301 (21.0)	7551 (20.8)	750 (22.8)
2–4	16 666 (42.2)	15 326 (42.3)	1340 (40.8)
5–7	13 633 (34.5)	12 535 (34.6)	1098 (33.4)
BMI (kg/m²)	26.5 (4.2)	26.3 (4.1)	28.5 (4.4)
DBP (mmHg)	81.4 (9.9)	81.3 (9.9)	82.5 (9.6)
SBP (mmHg)	135.0 (17.7)	134.5 (17.7)	140.6 (17.1)
Total serum cholesterol (mmol/L)	5.73 (1.08)	5.84 (1.03)	4.6 (0.89)
Diabetes	771 (2.0)	272 (0.8)	499 (15.2)
Stroke	160 (0.4)	66 (0.2)	94 (2.9)
CHD	820 (2.1)	162 (0.5)	658 (20.0)
Head injury	98 (0.3)	85 (0.2)	13 (0.4)
Depression	1782 (4.5)	1613 (4.5)	169 (5.1)
Insomnia			
Never/rarely	10 995 (27.8)	10 189 (28.1)	806 (24.5)
Sometimes	18 858 (47.7)	17 346 (47.9)	1512 (46.0)
Usually	9632 (24.4)	8669 (23.9)	963 (29.3)
Statin type			
Simvastatin	2486 (6.3)		2486 (75.7)
Atorvastatin	569 (1.4)		569 (17.3)
Rosuvastatin	125 (0.3)		125 (3.8)
Pravastatin	96 (0.2)		96 (2.9)
Fluvastatin	10 (0.03)		10 (0.3)
Follow-up time (years)^a^	9.2 (2.7)	9.2 (2.7)	9.0 (2.7)
Outcomes			
GM volume (cm^3^)	614.9 (55.9)	616.0 (55.9)	602.6 (55.5)
WM volume (cm^3^)	546.0 (61.9)	545.6 (61.9)	550.6 (61.5)
Peripheral cortical GM volume (cm^3^)	479.3 (46.4)	480.2 (46.3)	469.3 (45.2)
WMH volume (cm^3^)^a^	2.9 (4.3)	2.7 (4.0)	4.9 (7.2)

^a^Median (interquartile range).

### Association of statin use with GM, WM, peripheral cortical GM and WMH volumes

We assessed the association of baseline statin use with total GM, total WM, peripheral cortical GM and WMH volumes measured 9 years later. Statin use was significantly associated with lower GM and peripheral cortical GM volumes across all three models (Table 2). In Model III, the mean difference was −1575 mm^3^ (−2358, −791) and −1448 mm^3^ (−2227, −668), respectively. Statin use also showed a significant positive association with WM volume in Model I. However, this association did not remain significant after further adjustment for lifestyle factors and health-related conditions (Models II and III). Finally, statin users manifested more WMH than statin non-users, even after adjustment for lifestyle factors and health-related conditions (Models II and III). In Model III, the mean difference (log-transformed) was 0.11 mm^3^ (0.07, 0.15), which is equivalent to 1.01 mm^3^ (0.97, 1.06) after back transformation.

**Table 2:** Association between statin use and global brain volumes

Volumes (mm^3^)		95% confidence interval			
	β	Lower	Upper	*P-*value	Adjusted *R*²
GM					
Model I	−3703	−4385	−3022	<0.0001	0.8925
Model II	−3563	−4253	−2874	<0.0001	0.8933
Model III	−1575	−2358	−791	<0.0001	0.8943
WM					
Model I	1100	421	1778	0.0015	0.9129
Model II	926	237	1614	0.0084	0.9132
Model III	−135	−919	649	0.7356	0.9135
Peripheral cortical GM					
Model I	−2676	−3351	−2001	<0.0001	0.8464
Model II	−2582	−3266	−1899	<0.0001	0.8475
Model III	−1448	−2227	−668	0.0003	0.8478
WMH (log-transformed)					
Model I	0.19	0.16	0.22	<0.0001	0.2774
Model II	0.18	0.15	0.21	<0.0001	0.2806
Model III	0.11	0.07	0.15	<0.0001	0.3007

β regression coefficient reflects the mean difference between groups in brain volume adjusting for the covariates mentioned in the model. Model I: adjusted for UK Biobank imaging centre, age, sex, ethnicity, education, TDI, *APOE4* status, antidepressant use and intracranial volume. Model II: adjusted for all covariates in Model I and lifestyle factors (alcohol intake frequency, smoking and physical activity). Model III adjusted for all covariates in Model II and health-related conditions (BMI, SPB, DPB, diabetes, CHD, stroke, head injury, depression and insomnia).

**Figure 2:**
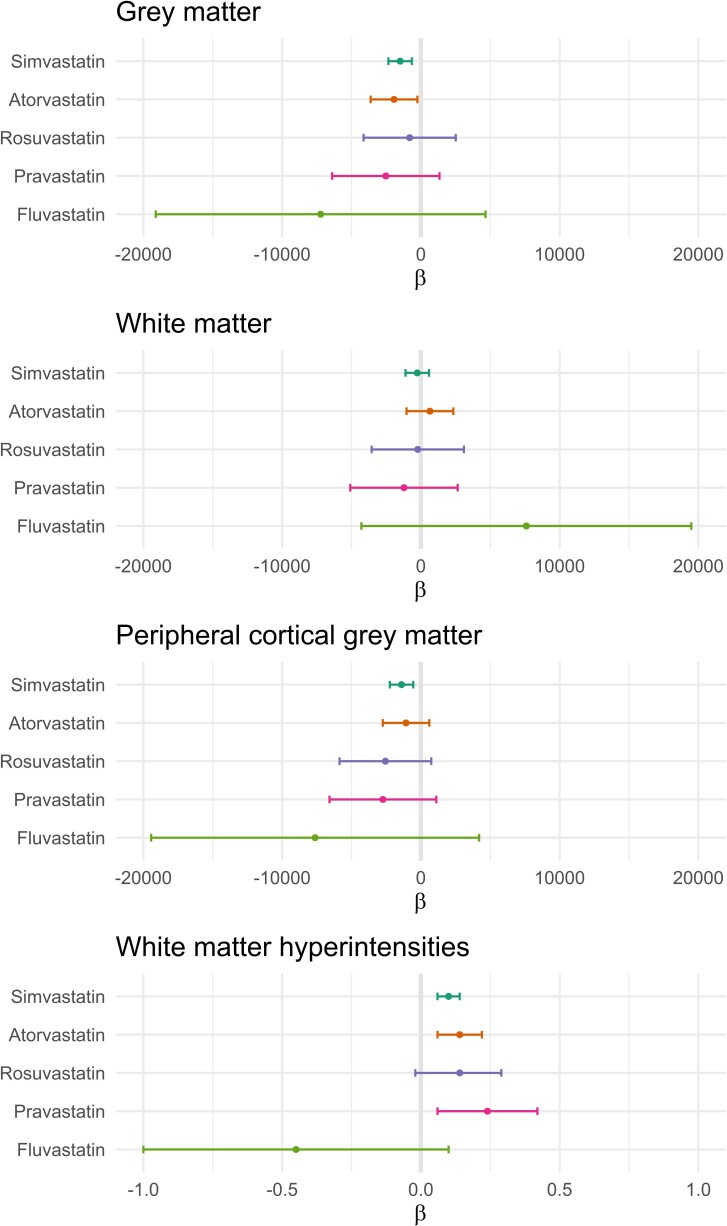
**Associations between statin type and the volumes of GM, WM, peripheral cortical GM and WMH.** The number of users for each statin was 2486 for simvastatin; 569 for atorvastatin; 125 for rosuvastatin; 96 for pravastatin; and 10 for fluvastatin. Linear models were adjusted for UK Biobank imaging centre, age, sex, ethnicity, education, TDI, *APOE4* status, antidepressant use, intracranial volume, lifestyle factors (alcohol intake frequency, smoking and physical activity) and health-related conditions (BMI, SBP, DBP, diabetes, CHD, stroke, head injury, depression and insomnia). The significance of the β estimates of statin type was evaluated using *t* statistic with 36 839 degrees of freedom.

Model III was replicated according to statin type. Simvastatin and atorvastatin were observed to be the most commonly prescribed statins and were negatively associated with GM volume (Fig. 2) with a mean difference of −1495 mm^3^ (−2342, −647) and −1934 mm^3^ (−3618, −249), respectively (Supplementary Table 1). None of the statins showed a significant association with WM volume, and simvastatin was the only statin associated with lower peripheral cortical GM volume [β = −1392 mm^3^ (−2234, −549)]. Finally, the use of simvastatin, atorvastatin and pravastatin was significantly associated with higher WMH.

### Mediation of total serum cholesterol in the association between statin use and global brain volumes

**Figure 3:**
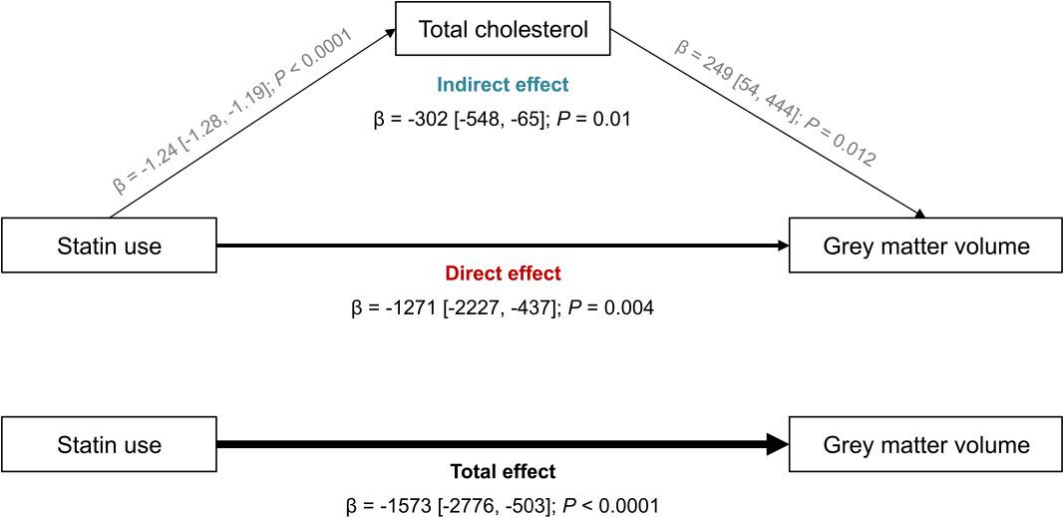
**The association between statin use and GM volume is mediated by total serum cholesterol.** The figure illustrates the mediation pathway, highlighting the role of total cholesterol in the relationship between statin use and GM volume. Arrows indicate the direction of influence, providing insights into the potential mediating effect of total cholesterol. All the models were adjusted for UK Biobank imaging centre, age, sex, ethnicity, education, TDI, *APOE4* status, antidepressant use, intracranial volume, lifestyle factors (alcohol intake frequency, smoking and physical activity) and health-related conditions (BMI, SBP DBP, diabetes, CHD, stroke, head injury, depression and insomnia). The average direct effect, indirect effect and total effect and their confidence intervals were computed using mediation package in R with 1000 simulations.

To investigate whether total serum cholesterol mediated the association between statin use and global brain volumes, mediation analyses were conducted. First, we assessed the association between statin use and cholesterol. Statin use was found to be associated with a reduction of 1.24 mmol/L (1.28, 1.19) in cholesterol concentration (Fig. 3). Second, we evaluated the association between total serum cholesterol concentration and GM volume after controlling for statin use and Model III covariates. A one-unit increase in cholesterol concentration was associated with higher GM volume [β = 249 mm^3^ (54, 444)]. Finally, we computed the average direct, indirect and total effects, as well as the proportion mediated. Overall, total serum cholesterol significantly mediated the association between statin use and GM volume with an indirect effect of −302 mm^3^ (−548, −65). The proportion mediated was 20%. However, no mediation effect of total serum cholesterol was observed for the association of statin use with WM, peripheral cortical GM and WMH (Supplementary Table 2).

### Association of statin use with cortical and subcortical brain volumes

**Figure 4:**
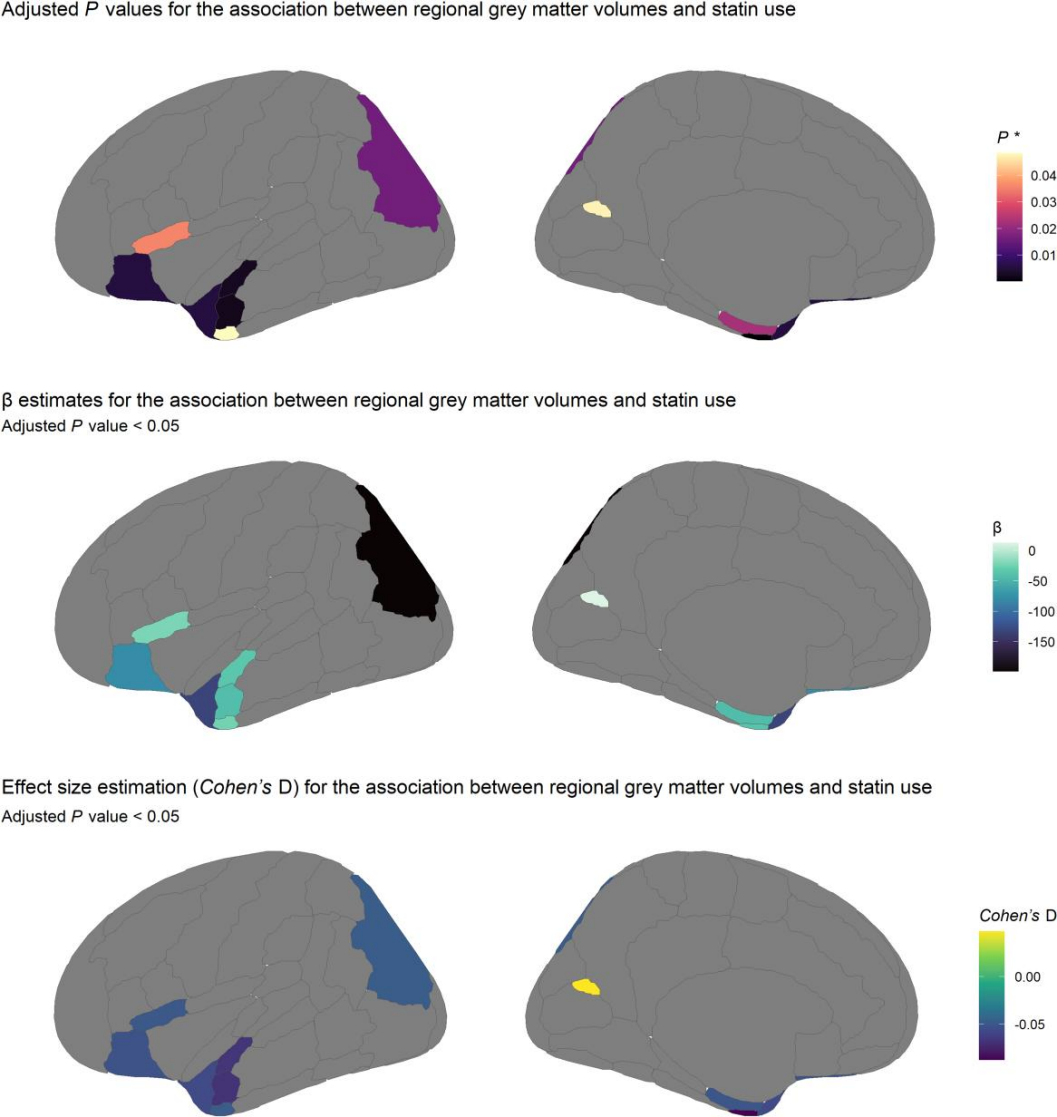
**Regional GM volumes significantly associated with statin use.** The figure shows a graphical representation of the GM regions on the lateral (left) and medial (right) sides. The model was adjusted for UK Biobank imaging centre, age, sex, ethnicity, education, TDI, *APOE4* status, antidepressant use, intracranial volume, lifestyle factors (alcohol intake frequency, smoking and physical activity) and health-related conditions (BMI, SBP, DBP, diabetes, CHD, stroke, head injury, depression and insomnia). *P-*values were obtained using *t* statistic with 36 839 degrees of freedom and corrected for multiple testing (*P* *). β estimates reflect the mean volume difference between statin users and non-users adjusting for the covariates mentioned in the model. For each regional volume, Cohen’s *D* was calculated by dividing β by the standard deviation.

In the secondary analysis, we further explored the association between baseline statin use and 48 cortical volumes and seven subcortical GM volumes (measured 9 years later). After correction for multiple testing, 14 regions were significantly associated with statin use in Model III (Supplementary Table 3). Compared with non-users, statin users demonstrated smaller subcortical volumes in thalamus [β = −102 mm^3^ (−142, −61)], pallidum [β = −22 mm^3^ (−39, −5)], hippocampus [β = −43 mm^3^ (−76, −10)] and nucleus accumbens [β = −18 mm^3^ (−26, −10)]. Statin use was also significantly associated with changes in 10 cortical GM regions (Fig. 4). Statin users demonstrated smaller volumes in nine cortical GM regions with the highest effect size for the temporal fusiform cortex anterior (Cohen’s *D* = −0.09), the middle temporal gyrus anterior (Cohen’s *D* = −0.07) and the superior temporal gyrus anterior (Cohen’s *D* = −0.07). In contrast, statin use was associated with a larger volume in the supracalcarine cortex (Cohen’s *D* = 0.05).

Overall, statin use was associated with lower GM volumes in the anterior temporal region and parts of the frontal and occipital brain regions (Supplementary Table 3).

### Sensitivity analysis

A first sensitivity analysis was conducted to specifically assess the association between long-term statin use and global brain volumes. All the results of the sensitivity analysis were consistent with the findings in the main analysis (Table 3). Compared with non-users, long-term statin use was significantly associated with lower GM [β = −1796mm^3^ (−2693, −898)] and peripheral cortical GM volumes [β = −1380 mm^3^ (−2276, −484)] and higher WMH [β = 0.10 mm^3^ (0.06, 0.14)].

**Table 3:** Association between long-term statin use and global brain volumes

Volumes (mm^3^)		95% confidence interval			
	β	Lower	Upper	*P-*value	Adjusted *R*²
GM					
Model I	−3647	−4422	−2872	<0.0001	0.896
Model II	−3455	−4239	−2671	<0.0001	0.8969
Model III	−1796	−2693	−898	<0.0001	0.8977
WM					
Model I	1173	402	1945	0.0029	0.9158
Model II	974	191	1756	0.0147	0.916
Model III	60	−838	958	0.8954	0.9163
Peripheral cortical GM					
Model I	−2375	−3146	−1603	<0.0001	0.8508
Model II	−2234	−3014	−1454	<0.0001	0.8518
Model III	−1380	−2276	−484	0.0025	0.8521
WMH (log-transformed)					
Model I	0.17	0.13	0.21	<0.0001	0.2749
Model II	0.16	0.12	0.2	<0.0001	0.2773
Model III	0.10	0.06	0.14	<0.0001	0.2956

β regression coefficient reflects the mean difference between groups in brain volume adjusting for the covariates mentioned in the model. Model I: adjusted for UK Biobank imaging centre, age, sex, ethnicity, education, TDI, *APOE4* status, antidepressant use and intracranial volume. Model II: adjusted for all covariates in Model I and lifestyle factors (alcohol intake frequency, smoking and physical activity). Model III adjusted for all covariates in Model II and health-related conditions (BMI, SPB, DPB, diabetes, CHD, stroke, head injury, depression and insomnia).

A second sensitivity analysis was performed to account for recall bias in self-reported health-related conditions (diabetes, CHD, stroke, head injury, depression and insomnia). After replacing self-reported conditions with ICD-10 diagnoses, we found similar results compared with the main analysis (Supplementary Table 4).

## Discussion

The current study investigates the associations between statin use and brain volumes among 39 514 middle-aged and old participants from the UK Biobank cohort. Statin use was found to be associated with lower GM volume and higher WMH measured 9 years later. These associations persisted after adjustment for demographic characteristics, lifestyle factors and health-related conditions. Similar associations were found among long-term statin users. Moreover, the association between statin use and GM volume was partially mediated by total serum cholesterol concentration (20% of the effect). Secondary analysis demonstrated reduced volumes among statin users in four subcortical and nine cortical GM regions. Overall, statin use was associated with lower GM volumes in the anterior temporal region and parts of the frontal and occipital regions.

We found that statin users displayed lower GM, lower peripheral cortical GM and higher WMH, while the association between statin use and WM volume was inconsistent according to the different models. In line with our results, a recent study calculated the 4-year difference in WMH volume and found greater WMH progression in statin users compared with non-users.^16^ Counter to this, some randomized controlled trials have shown that statin use was associated with lower progression of WMH among individuals with severe WMH at baseline,^17^ with poor cognitive status,^37^ or in older hypertensive patients.^21,22^ In another randomized controlled trial of healthy middle-aged adults with an 18-month follow-up, no significant change in GM volume was found, while changes in WM were slightly higher in the simvastatin group compared with placebo. Besides, changes in WM microstructure were identified with increased fractional anisotropy and decreased radial and mean diffusivity in the simvastatin group.^20^ However, this study and two others reported no effect of statin on the difference in WMH volume.^19,20,38^

To our knowledge, this study is the first to evaluate the association between statin use and specific brain regions measured with FAST and FIRST brain scan segmentation. In particular, statin use was found to be associated with lower volume of the hippocampus and the anterior parahippocampal gyrus 9 years later. Conversely, a case–cohort study of 33 individuals reported no hippocampal volume change after 2 years of statin therapy.^23^ In addition, a 2-year follow-up MRI study did not report significant changes in these brain areas between statin ever-users and never-users, but the authors found that parahippocampal volume decline was attenuated among statin users with diabetes.^18^ Overall, comparing the findings of the present study with these previous studies is challenging due to the difference in study samples, follow-up periods and methodologies. More specifically, the population enrolled were either older adults,^18,19,23,37–39^ individuals with risk factors for cardiovascular events^16,17^ or those with a parental family history of Alzheimer’s disease,^20^ whereas the population of the present study was predominantly comprised of healthy middle-aged and older participants.^40^ Second, some of these studies had relatively few participants (Doraiswamy *et al.*^23^  *n* = 33, Vogt *et al.*^20^  *n* = 72, Mok *et al.*^17^  *n* = 208, Goldstein *et al.*^16^  *n* = 425, Samaras *et al.*^18^  *n* = 526, ten Dam *et al.*^19^  *n* = 535, Ji *et al.*^21^  *n* = 668 and Zhang *et al.*^22^  *n* = 732), which limits their statistical power and constrains the generalizability of the results. Third, some studies, similar to ours, performed cross-sectional analysis with single brain imaging examination and included a longitudinal component of statin use,^37,38^ while others were longitudinal with several time points of brain imaging.^16,18,23,39^ Finally, other significant limitations also exist in the available literature, including a lack of adjustment for important covariates, especially *APOE4* status.^16,17,19,22^

Simvastatin and atorvastatin were found to be associated with lower GM but not the other brain volumes. This finding is not surprising because they are also the most frequently prescribed statins in the UK Biobank, with 2486 and 569 participants, respectively. However, the association between statin use and WMH was also evident pravastatin (*n* = 96), which is a hydrophilic statin. These findings suggest that both lipophilic and hydrophilic statins may promote WMH. However, the association between fluvastatin use and brain volumes remains inconclusive, likely due to the low number of participants (*n* = 10).

The findings of mediation analysis showed that total serum cholesterol concentration partially mediated the association between statin use and GM volume. These findings suggest that cholesterol reduction through statin use contributes to reduced GM volume. We hypothesize that cholesterol concentration and GM volume might be related by a U-shape association, wherein both low cholesterol and high cholesterol have been associated with reduced cognition.^41^ This association may also vary across lifespan, with high total cholesterol concentration in midlife associated with higher dementia risk, but not in late life.^42^ This emphasizes the importance of early prevention of cardiovascular risk factors to promote long-term brain health.^43^

Statins may also exert their action directly within brain cells. Indeed, statins have been detected in the cerebral cortex of mice 6 h after their administration, irrespective of lipophilic properties,^44^ supporting their ability to cross the blood–brain barrier. Plasma 24S-hydroxycholesterol, used as a marker of brain cholesterol turnover, is produced by CYP46A (cytochrome P450 family 46 subfamily A) in the brain by cholesterol hydroxylation and is excreted in the blood.^9,45,46^ After statin treatment, some studies have demonstrated a reduction in plasma 24S-hydroxycholesterol concentration in guinea pigs^11^ and in patients with Alzheimer’s disease.^10^ These findings suggest that statins may reduce brain cholesterol metabolism.

The major strengths of this study include the large number of participants, which is larger than in all previous studies that addressed the same research question. Moreover, the analysis was adjusted for *APOE4* status and history of CHD, stroke and diabetes that could have affected brain volumes over time and statin therapy initiation.^2,7,15^ Furthermore, the associations between statin use and specific brain regions were comprehensively analysed and corrected for multiple testing. However, this study presents some limitations that should be acknowledged. First, we performed a cross-sectional analysis of a prospective cohort with statin use assessed twice (baseline and imaging visit) and brain volume assessed once (imaging visit). Because baseline brain MRI was not performed in the UK Biobank, we could not evaluate the longitudinal association between statin use and brain volume changes. Besides, it is possible that the brain alterations were already presented before statin exposure. To minimize this bias, we excluded all subjects with medical conditions that could significantly affect brain volumes. Second, information regarding statin dosage and precise duration of statin use before the enrolment was not available in the UK Biobank. Thus, we performed a sensitivity analysis to specifically assess the association between participants who kept using statin between the baseline and the imaging visit (i.e. for a median of 9 years) and participants who never used statin during the same period.

## Conclusion

The study findings suggest a significant association of statin use over a long time with lower GM volume and higher WMH. Moreover, this study is the first to identify potential associations of statin use with smaller subcortical brain volumes, such as thalamus, nucleus accumbens and hippocampus, and smaller cortical brain volumes in the temporal lobe region. To confirm and expand these findings, this analysis should be replicated in longitudinal studies with multiple brain imaging examinations and information on statin dosage and duration. This study also revealed that statin-induced reduction in total serum cholesterol is involved in GM alteration. Overall, this research emphasizes the differential effects of statin use on brain structure, with potential direct and indirect effects.

## Supplementary Material

fcae417_Supplementary_Data

## Data Availability

This project was conducted using the UK Biobank resource under application number 30172. All the data that support the findings of this study are available from the UK Biobank. Permissions are required to gain access to the UK Biobank data resources, subject to successful registration and application process. Further information can be found on the UK Biobank website (www.ukbiobank.ac.uk). Codes generated to analyse the data are provided in the Supplementary material.
