# Supplementary material for: Statin use is associated with higher white matter hyperintensity volumes and lower grey matter volumes
Source: Brain Commun. 2024 Nov 20;6(6):fcae417. doi: 10.1093/braincomms/fcae417 (PMC11606650; doi:10.1093/braincomms/fcae417)
Supplement: fcae417_Supplementary_Data [file fcae417_supplementary_data.pdf]

Supplementary Table 1. Association between statin type and global brain volumes.

Volume (mm^3)	Statin type	β	95% Confidence Interval Lower CI	Upper CI	*P* value	Adjusted R^2
**Grey matter**	No statin	Ref	-	-	-	0.8943
	Simvastatin	-1495	-2342	-647	0.0005	
	Atorvastatin	-1934	-3618	-249	0.0244	
	Rosuvastatin	-808	-4131	2515	0.6338	
	Pravastatin	-2527	-6396	1342	0.2004	
	Fluvastatin	-7222	-19107	4663	0.2337	
**White matter**	No statin	Ref	-	-	-	0.9135
	Simvastatin	-261	-1108	587	0.5465	
	Atorvastatin	654	-1032	2339	0.4471	
	Rosuvastatin	-221	-3546	3104	0.8962	
	Pravastatin	-1220	-5091	2651	0.5369	
	Fluvastatin	7604	-4288	19495	0.2101	
**Peripheral cortical grey matter**	No statin	Ref	-	-	-	0.8478
	Simvastatin	-1392	-2234	-549	0.0012	
	Atorvastatin	-1068	-2743	608	0.2117	
	Rosuvastatin	-2557	-5862	749	0.1295	
	Pravastatin	-2736	-6584	1112	0.1635	
	Fluvastatin	-7621	-19442	4200	0.2064	
**WMH (log-transformed)**	No statin	Ref	-	-	-	0.3008
	Simvastatin	0.10	0.06	0.14	<0.0001	
	Atorvastatin	0.14	0.06	0.22	0.0005	
	Rosuvastatin	0.14	-0.02	0.29	0.0888	
	Pravastatin	0.24	0.06	0.42	0.0095	
	Fluvastatin	-0.45	-1.0	0.10	0.1086	

Models were adjusted for UK Biobank imaging centre, age, sex, ethnicity, education, Townsend deprivation index, *APOE4* status, antidepressant use, intracranial volume, lifestyle factors (alcohol intake frequency, smoking, physical activity), and health-related conditions (body mass index, systolic and diastolic blood pressure, diabetes, coronary heart disease, stroke, head injury, depression, insomnia).

Supplementary Table 2. The mediation of total cholesterol in the association between statin use and volumes of grey matter, white matter, peripheral cortical grey matter and white matter hyperintensities.

**Volume (mm^3)**	**Total effect** β (CI)	**Direct effect** β (CI)	**Indirect effect** β (CI)	**Proportion mediated**	***P* value of the mediated effect**
**Grey matter**	-1573 (-2776,-503)	-1271 (-2227,-437)	-302 (-548,-65)	20%	0.012
**White matter**	-103 (-1220,1108)	-307 (-1200,649)	204 (-19,459)	-13%	0.87
**Peripheral cortical grey matter**	-1373 (-2538,-224)	-1218 (-2144,-319)	-155 (-394,95)	11%	0.204
**WMH (log-transformed)**	0.12 (0.07,0.17)	0.11 (0.07,0.15)	0.01 (0,0.02)	7 %	0.172

All the models within the analysis were adjusted for UK Biobank imaging centre, age, sex, ethnicity, education, Townsend deprivation index, *APOE4* status, antidepressant use, intracranial volume, lifestyle factors (alcohol intake frequency, smoking, physical activity), and health-related conditions (body mass index, systolic and diastolic blood pressure, diabetes, coronary heart disease, stroke, head injury, depression, insomnia).

.

Supplementary Table 3. Associations between statin use and regional grey matter volumes.

Volumes (mm^3)	β	95% Confidence Interval		*P* value	Adjusted *P*
		**Lower**	**Upper**		
**Thalamus**	-102	-142	-61	9.38E-07	**3.00E-05**
Caudate nucleus	27	-3	56	0.0758	0.1545
Putamen	-9	-45	27	0.6306	0.7078
**Pallidum**	-22	-39	-5	0.0122	**0.0483**
**Hippocampus**	-43	-76	-10	0.01	**0.0472**
Amygdala	-21	-38	-4	0.0182	0.0572
**Nucleus accumbens**	-18	-26	-10	4.12E-06	**7.56E-05**
**Temporal Pole**	-134	-211	-57	0.0006	**0.0054**
**Superior Temporal Gyrus, anterior division**	-35	-53	-16	0.0002	**0.0024**
Superior Temporal Gyrus, posterior division	-7	-37	24	0.6654	0.7126
**Middle Temporal Gyrus, anterior division**	-43	-65	-21	0.0001	**0.0020**
Middle Temporal Gyrus, posterior division	-15	-65	36	0.5714	0.6687
Middle Temporal Gyrus, temporooccipital part	-21	-73	31	0.4201	0.5374
**Inferior Temporal Gyrus, anterior division**	-24	-44	-5	0.0123	**0.0483**
Inferior Temporal Gyrus, posterior division	11	-37	59	0.6515	0.7126
Inferior Temporal Gyrus, temporooccipital part	18	-25	60	0.4165	0.5374
**Parahippocampal Gyrus, anterior division**	-43	-72	-14	0.0036	**0.0223**
Parahippocampal Gyrus, posterior division	-8	-22	6	0.2427	0.3813
**Temporal Fusiform Cortex, anterior division**	-38	-53	-23	1.09E-06	**3.00E-05**
Temporal Fusiform Cortex, posterior division	-2	-34	30	0.9032	0.9062
Temporal Occipital Fusiform Cortex	24	-8	56	0.1482	0.2629
Planum Polare	-9	-23	4	0.1842	0.3167
Heschl's Gyrus (includes H1 and H2)	-14	-29	0	0.0567	0.1300
Planum Temporale	-11	-36	13	0.3549	0.5028
Superior Frontal Gyrus	-23	-131	85	0.6738	0.7126
Middle Frontal Gyrus	120	16	225	0.0243	0.0669
Frontal Gyrus, pars opercularis	-32	-65	1	0.0594	0.1308
Frontal Gyrus, pars triangularis	-14	-49	21	0.4328	0.5410
**Frontal Orbital Cortex**	-80	-127	-34	0.0007	**0.0054**
Precentral Gyrus	-128	-235	-21	0.0187	0.0572
Frontal Medial Cortex	-11	-34	13	0.3656	0.5028
Frontal Pole	-61	-204	82	0.4046	0.5374
Subcallosal Cortex	-23	-47	0	0.0546	0.1300
**Frontal Operculum Cortex**	-22	-38	-6	0.0065	**0.0357**
Postcentral Gyrus	-42	-134	49	0.3630	0.5028
Superior Parietal Lobule	-49	-107	9	0.0972	0.1843
Supramarginal Gyrus, anterior division	19	-20	59	0.3447	0.5028
Supramarginal Gyrus, posterior division	19	-39	76	0.5211	0.6369
Angular Gyrus	40	-20	99	0.1921	0.3201
Intracalcarine Cortex	-3	-46	41	0.9062	0.9062
Precuneous Cortex	-54	-137	29	0.2006	0.3244
Parietal Operculum Cortex	-8	-35	19	0.5567	0.6656
Lingual Gyrus	-56	-108	-5	0.0318	0.0833
**Lateral Occipital Cortex, superior division**	-199	-327	-71	0.0023	**0.0160**

Volumes (mm^3)	95% Confidence Interval				
	**β**	**Lower**	**Upper**	***P* value**	**Adjusted *P***
Lateral Occipital Cortex, inferior division	7	-66	80	0.8549	0.8871
Cuneal Cortex	-8	-37	21	0.6039	0.6919
Occipital Fusiform Gyrus	-33	-69	3	0.0747	0.1545
**Supracalcarine Cortex**	12	3	22	0.0103	**0.0472**
Occipital Pole	-70	-157	16	0.1114	0.2042
Paracingulate Gyrus	-66	-120	-12	0.0170	0.0572
Cingulate Gyrus, anterior division	69	-9	147	0.0837	0.1644
Cingulate Gyrus, posterior division	47	2	91	0.0392	0.0980
Insular Cortex	-50	-90	-10	0.0151	0.0553
Central Opercular Cortex	-39	-73	-6	0.0223	0.0645
Juxtapositional Lobule Cortex	-22	-62	19	0.3032	0.4632

Linear models were adjusted for UK Biobank imaging centre, age, sex, ethnicity, education, Townsend deprivation index, *APOE4* status, antidepressant use, intracranial volume, lifestyle factors (alcohol intake frequency, smoking, physical activity), and health-related conditions (body mass index, systolic and diastolic blood pressure, diabetes, coronary heart disease, stroke, head injury, depression, insomnia). *P* values were corrected for multiple testing (Adjusted *P*). β estimates reflect the mean volume difference between statin users and non-users adjusting for the covariates mentioned in the model.

Supplementary Table 4. Association between statin use and global brain volumes after adjustment for medical history based on ICD-10 diagnoses.

Volume (mm^3)		95% Confidence Interval			
	**β**	**Lower CI**	**Upper CI**	***P* value**	**Adjusted R^2**
**Grey matter**	-2597	-3342	-1851	<0.0001	0.894
**White matter**	223	-523	968	0.5583	0.9135
**Peripheral cortical grey matter**	-2304	-3045	-1563	<0.0001	0.8475
**WMH (log-transformed)**	0.14	0.11	0.18	<0.0001	0.3001

Models were adjusted for UK Biobank imaging centre, age, sex, ethnicity, education, Townsend deprivation index, *APOE4* status, antidepressant use, intracranial volume, lifestyle factors (alcohol intake frequency, smoking, physical activity), and health-related conditions (body mass index, systolic and diastolic blood pressure, and diabetes, coronary heart disease, stroke, head injury, depression, insomnia reported based on ICD-10 diagnoses).

Statin use and brain volume alterations

Mélissa Gentreau

2024-06-24

This code requires the packages *tidyverse*, *knitr*, *png*, *ggplot2*, *RColorBrewer*, *cowplot*, and *mediation*.

Function used to create the result table:

```
multivarlmR2 <- function (data, outcome, varexpo, varajust,
                          labelexpo = varexpo, decimal = 2) {
  # data        dataset
  # outcome     character vector of outcome names
  # varexpo     character vector of exposure variable (1)
  # varajust    character vector of covariates
  # labelexpo   character vector of labels of the exposure variable, colnames by default
  # decimal     number of digits after the decimal point, 2 by default

  x <- data[,c(varexpo)]
  res <- NULL

  for (i in 1:length(outcome)) {
    # y : variable i to explain
    y <- outcome[i]

    ## Empty matrix which will be used as a table
    mat_res <- matrix(NA, nrow = 0, ncol = 9)
    colnames(mat_res) <- c("Outcome","Exposure","Beta","SE",
                           "Lower CI", "Upper CI" ,"P value", "P.raw",
                           "Adjusted R-squared")

    varexpo <- c(varexpo)

    data2 <- na.omit(data[, c(y, varexpo, varajust)])


    # Writing the equation y ~ x1 + x2 +...+ xp
    formule <- paste(y, "~", varexpo)
    for (k in 1:length(varajust)) {formule <- paste(formule, "+", varajust[k])}
    formule <- as.formula(formule)

    # Model
    mod <- lm(formula = formule, data = data2)

    # Display results of the exposure variable only
```

```r
  # i.e. the second row of coefficients and ICs (the first being the intercept)

  # Coefficients and standard error
  beta <- round(mod$coefficients, decimal)
  se <- round(summary(mod)$coefficients[, 2], decimal)
  CI <- round(confint(mod), decimal)

  # P-value extraction
  pval <- summary(mod)$coefficients[, 4]
  pvalr <- round(pval, 4)
  pvalr[as.double(pval) < 0.0001] <- "<0.0001"

  # Adjusted R-squared value
  adj.r.squared <- round(summary(mod)$adj.r.squared, 4)

  # If the exposure variable is a factor
  if (is.factor(x)) {

    lev <- levels(x)
    nlev <- nlevels(x)

    if (nlev == 2){
      line <- c(y,
                paste(varexpo, "_", lev[2], sep = ""),
                beta[2], se[2],
                CI[2,1], CI[2,2], pvalr[2], pval[2], adj.r.squared)
      mat_res <- rbind(mat_res, line)

    } else if (nlev > 2) {
      line0 <- c(y, paste(varexpo, "_", lev[1], sep = ""), rep("", 6), adj.r.squared)
      mat_res <- rbind(mat_res, line0)

      for (k in 2:nlev) {
        line <- c(y,
                  paste(varexpo, "_", lev[k], sep = ""),
                  beta[k], se[k],
                  CI[k,1], CI[k,2], pvalr[k], pval[k], "")
        mat_res <- rbind(mat_res, line)
      }
    }

  } else if (is.numeric(x)){

    line <- c(y, varexpo, beta[2], se[2],
              CI[2,1], CI[2,2], pvalr[2], pval[2], adj.r.squared)
    mat_res <- rbind(mat_res, line)
  }

  res <- rbind(res, mat_res)
  row.names(res) <- NULL

}
```

```
  return(res)

}
```

MAIN ANALYSES

```
imagsel <- c("GM_vol2", "WM_vol2", "cortical_vol2", "logWMH_vol2")

# Model 1
mmd1 <- multivarlmR2(data = as.data.frame(ukb2),
                    outcome = c(imagsel),
                    varexpo = "statin0b",
                    varajust = c("centre2","age0","sex","ethnic0b","qualif0b",
                                 "TDI0","APOE4","antidep2","ICV2"),
                    decimal = 2)

mmd1 <- as.data.frame(mmd1)
mmd1$Model <- rep("I",4)

# Model 2
mmd2 <- multivarlmR2(data = as.data.frame(ukb2),
                    outcome = c(imagsel),
                    varexpo = "statin0b",
                    varajust = c("centre2","age0","sex","ethnic0b","qualif0b",
                                 "TDI0","APOE4","antidep2","ICV2",
                                 "frq_alcohol0","smoking0","physact0b"),
                    decimal = 2)

mmd2 <- as.data.frame(mmd2)
mmd2$Model <- rep("II",4)

# Model 3
mmd3 <- multivarlmR2(data = as.data.frame(ukb2),
                    outcome = c(imagsel),
                    varexpo = "statin0b",
                    varajust = c("centre2","age0","sex","ethnic0b","qualif0b",
                                 "TDI0","APOE4","antidep2","ICV2",
                                 "frq_alcohol0","smoking0","physact0b",
                                 "BMI0","SBP0","DBP0","diabetes0","CHD0","stroke0",
                                 "headinjury0","depression0","insomn0"),
                    decimal = 2)

mmd3 <- as.data.frame(mmd3)
mmd3$Model <- rep("III",4)

res_mmd <- rbind(mmd1, mmd2, mmd3)
res_mmd <- res_mmd %>% arrange(Outcome)
res_mmd <- res_mmd[,-8]

res_mmd
```

```
##            Outcome   Exposure     Beta     SE Lower CI Upper CI P value
```

```
## 1        GM_vol2 statin0b_1 -3703.23 347.78 -4384.88 -3021.57 <0.0001
## 2        GM_vol2 statin0b_1 -3563.44 351.99 -4253.35 -2873.53 <0.0001
## 3        GM_vol2 statin0b_1 -1574.62 399.92 -2358.48  -790.76 <0.0001
## 4        WM_vol2 statin0b_1  1099.53 346.08   421.22  1777.85  0.0015
## 5        WM_vol2 statin0b_1   925.56 351.33   236.95  1614.18  0.0084
## 6        WM_vol2 statin0b_1  -135.14 400.14  -919.43   649.14  0.7356
## 7  cortical_vol2 statin0b_1 -2676.22  344.4 -3351.24 -2001.19 <0.0001
## 8  cortical_vol2 statin0b_1 -2582.38 348.74 -3265.92 -1898.84 <0.0001
## 9  cortical_vol2 statin0b_1 -1447.76 397.76 -2227.38  -668.13   3e-04
## 10   logWMH_vol2 statin0b_1     0.19   0.02     0.16     0.22 <0.0001
## 11   logWMH_vol2 statin0b_1     0.18   0.02     0.15     0.21 <0.0001
## 12   logWMH_vol2 statin0b_1     0.11   0.02     0.07     0.15 <0.0001
##    Adjusted R-squared Model
## 1              0.8925     I
## 2              0.8933    II
## 3              0.8943   III
## 4              0.9129     I
## 5              0.9132    II
## 6              0.9135   III
## 7              0.8464     I
## 8              0.8475    II
## 9              0.8478   III
## 10             0.2774     I
## 11             0.2806    II
## 12             0.3007   III
```

Model III according to statin type

```r
ukb2$statintype0 <- as.character(ukb2$statin0b)
ukb2$statintype0[ukb2$fluvastatin0 == "1"] <- "Fluvastatin"
ukb2$statintype0[ukb2$pravastatin0 == "1"] <- "Pravastatin"
ukb2$statintype0[ukb2$rosuvastatin0 == "1"] <- "Rosuvastatin"
ukb2$statintype0[ukb2$atorvastatin0 == "1"] <- "Atorvastatin"
ukb2$statintype0[ukb2$simvastatin0 == "1"] <- "Simvastatin"

ukb2$statintype0 <- factor(ukb2$statintype0,
                           levels = c("0","Simvastatin","Atorvastatin",
                                      "Rosuvastatin","Pravastatin","Fluvastatin"))
table(ukb2$statintype0)
```

```
##
##           0  Simvastatin Atorvastatin Rosuvastatin  Pravastatin  Fluvastatin
##       36217         2486          568          125           96           10
```

```r
m.type <- multivarlmR2(data = as.data.frame(ukb2),
                       outcome = c(imagsel),
                       varexpo = "statintype0",
                       varajust = c("centre2","age0","sex","ethnic0b","qualif0b",
                                    "TDI0","APOE4","antidep2","ICV2",
                                    "frq_alcohol0","smoking0","physact0b",
                                    "BMI0","SBP0","DBP0","diabetes0","CHD0","stroke0",
```

```r
                                     "headinjury0","depression0","insomn0"),
                        decimal = 2)

m.type <- as.data.frame(m.type)
m.type$Exposure <- rep(c("No statin","Simvastatin","Atorvastatin",
                         "Rosuvastatin","Pravastatin","Fluvastatin"), 4)
m.type <- m.type[,-8]
m.type
```

```
##            Outcome     Exposure     Beta      SE  Lower CI Upper CI P value
## 1          GM_vol2    No statin
## 2          GM_vol2  Simvastatin -1494.61  432.23  -2341.79  -647.43   5e-04
## 3          GM_vol2 Atorvastatin -1933.71  859.37  -3618.11  -249.31  0.0244
## 4          GM_vol2 Rosuvastatin  -807.58 1695.41  -4130.64  2515.48  0.6338
## 5          GM_vol2  Pravastatin -2527.38 1973.94  -6396.36  1341.61  0.2004
## 6          GM_vol2  Fluvastatin -7221.76 6063.78 -19106.94  4663.43  0.2337
## 7          WM_vol2    No statin
## 8          WM_vol2  Simvastatin  -260.76  432.45  -1108.38   586.87  0.5465
## 9          WM_vol2 Atorvastatin   653.72  859.83  -1031.56     2339  0.4471
## 10         WM_vol2 Rosuvastatin  -221.26  1696.3  -3546.06  3103.54  0.8962
## 11         WM_vol2  Pravastatin -1219.65 1974.98  -5090.67  2651.36  0.5369
## 12         WM_vol2  Fluvastatin   7603.7 6066.96  -4287.71 19495.12  0.2101
## 13 cortical_vol2    No statin
## 14 cortical_vol2  Simvastatin -1391.74  429.89  -2234.34  -549.14  0.0012
## 15 cortical_vol2 Atorvastatin -1067.58  854.73  -2742.86   607.71  0.2117
## 16 cortical_vol2 Rosuvastatin -2556.51 1686.24  -5861.59   748.58  0.1295
## 17 cortical_vol2  Pravastatin -2735.75 1963.27  -6583.81  1112.31  0.1635
## 18 cortical_vol2  Fluvastatin -7620.75 6030.99 -19441.66  4200.16  0.2064
## 19   logWMH_vol2    No statin
## 20   logWMH_vol2  Simvastatin      0.1    0.02      0.06     0.14 <0.0001
## 21   logWMH_vol2 Atorvastatin     0.14    0.04      0.06     0.22   5e-04
## 22   logWMH_vol2 Rosuvastatin     0.14    0.08     -0.02     0.29  0.0888
## 23   logWMH_vol2  Pravastatin     0.24    0.09      0.06     0.42  0.0095
## 24   logWMH_vol2  Fluvastatin    -0.45    0.28        -1      0.1  0.1086
##    Adjusted R-squared
## 1              0.8943
## 2
## 3
## 4
## 5
## 6
## 7              0.9135
## 8
## 9
## 10
## 11
## 12
## 13             0.8478
## 14
## 15
## 16
## 17
## 18
```

```
## 19             0.3008
## 20
## 21
## 22
## 23
## 24
```

```r
m.type.p <- subset(m.type, m.type$Exposure != "No statin")
colnames(m.type.p)[5:7] <- c("Lower_CI","Upper_CI","P")
m.type.p$Beta <- as.numeric(m.type.p$Beta)
m.type.p$Lower_CI <- as.numeric(m.type.p$Lower_CI)
m.type.p$Upper_CI <- as.numeric(m.type.p$Upper_CI)
m.type.p$Exposure <- factor(m.type.p$Exposure,
                            levels = c("Fluvastatin","Pravastatin","Rosuvastatin",
                                       "Atorvastatin","Simvastatin"))


p1 <- m.type.p %>% subset(m.type.p$Outcome == "GM_vol2") %>%
  ggplot(aes(x = Exposure , y = Beta, color = Exposure)) +
  geom_point(size = 0.9) +
  geom_hline(yintercept = 0, lty = 1, lwd = 1, color = "grey90") +
  geom_errorbar(aes(ymin = Lower_CI, ymax = Upper_CI), width=.2) +
  scale_color_discrete(type = rev(brewer.pal(n = 5, name = "Dark2"))) +
  xlab("") +
  ylab(expression(Beta)) +
  ylim(c(-20000,20000)) +
  ggtitle("Grey matter") +
  coord_flip() +
  guides(fill = "none", color = "none", linetype = "none", shape = "none") +
  theme_minimal()


p2 <- m.type.p %>% subset(m.type.p$Outcome== "WM_vol2") %>%
  ggplot(aes(x = Exposure, y = Beta, color = Exposure)) +
  geom_point(size = 0.9) +
  geom_hline(yintercept = 0, lty = 1, lwd = 1, color = "grey90") +
  geom_errorbar(aes(ymin = Lower_CI, ymax = Upper_CI), width= 0.2) +
  scale_color_discrete(type = rev(brewer.pal(n = 5, name = "Dark2"))) +
  xlab("") +
  ylab(expression(Beta)) +
  ylim(c(-20000,20000)) +
  ggtitle("White matter") +
  coord_flip() +
  guides(fill = "none", color = "none", linetype = "none", shape = "none") +
  theme_minimal()

p3 <- m.type.p %>% subset(m.type.p$Outcome == "cortical_vol2") %>%
  ggplot(aes(x = Exposure, y = Beta, color = Exposure)) +
  geom_point(size = 0.9)+
  geom_hline(yintercept = 0, lty = 1, lwd = 1, color = "grey90") +
  geom_errorbar(aes(ymin = Lower_CI, ymax = Upper_CI), width=.2) +
```

```r
  scale_color_discrete(type = rev(brewer.pal(n = 5, name = "Dark2"))) +
  xlab("") +
  ylab(expression(Beta)) +
  ylim(c(-20000,20000)) +
  ggtitle("Peripheral cortical grey matter") +
  coord_flip() +
  guides(fill = "none", color = "none", linetype = "none", shape = "none") +
  theme_minimal()


p4 <- m.type.p %>% subset(m.type.p$Outcome == "logWMH_vol2") %>%
  ggplot(aes(x = Exposure, y = Beta, color = Exposure)) +
  geom_point(size = 0.9)+
  geom_hline(yintercept = 0, lty = 1, lwd = 1, color = "grey90") +
  geom_errorbar(aes(ymin = Lower_CI, ymax = Upper_CI), width = .2) +
  scale_color_discrete(type = rev(brewer.pal(n = 5, name = "Dark2"))) +
  xlab("") +
  ylab(expression(Beta)) +
  ylim(c(-1,1)) +
  ggtitle("White matter hyperintensities") +
  coord_flip() +
  guides(fill = "none", color = "none", linetype = "none", shape = "none") +
  theme_minimal()

# plot_grid(p1, p2, p3, p4, ncol = 1)
```

Figure: Associations between statin type and the volumes of grey matter, white matter, peripheral cortical grey matter and white matter hyperintensity.

MEDIATION ANALYSIS

Statin, total cholesterol, and grey matter

```r
GM <- lm(GM_vol2 ~ statin0b + chol0 + centre2 + age0 + sex + ethnic0b + qualif0b +
           TDI0 + APOE4 + antidep2 + ICV2 + frq_alcohol0 + smoking0 + physact0b +
           BMI0 + SBP0 + DBP0 + diabetes0 + CHD0 + stroke0 + headinjury0 +
           depression0 + insomn0,
         data = ukb2)

GM.med1 <- lm(chol0 ~ statin0b + centre2 + age0 + sex + ethnic0b + qualif0b +
           TDI0 + APOE4 + antidep2 + ICV2 + frq_alcohol0 + smoking0 + physact0b +
             BMI0 + SBP0 + DBP0 + diabetes0 + CHD0 + stroke0 + headinjury0 +
             depression0 + insomn0,
              data = ukb2)


GM.chol <- mediate(GM.med1, GM, treat = "statin0b", mediator = "chol0",
                      robustSE = T, sims = 1000)

summary(GM.chol)
```

```
##
## Causal Mediation Analysis
##
## Quasi-Bayesian Confidence Intervals
##
##                  Estimate 95% CI Lower 95% CI Upper p-value
## ACME            -3.02e+02    -5.48e+02       -65.38   0.012 *
## ADE             -1.27e+03    -2.23e+03      -437.46   0.004 **
## Total Effect    -1.57e+03    -2.48e+03      -769.63  <2e-16 ***
## Prop. Mediated   1.95e-01     4.31e-02         0.47   0.012 *
## ---
## Signif. codes:  0 '***' 0.001 '**' 0.01 '*' 0.05 '.' 0.1 ' ' 1
##
## Sample Size Used: 35222
##
##
## Simulations: 1000
```

Statin, total cholesterol, and white matter

```
WM <- lm(WM_vol2 ~ statin0b + chol0 + centre2 + age0 + sex + ethnic0b + qualif0b +
           TDI0 + APOE4 + antidep2 + ICV2 + frq_alcohol0 + smoking0 + physact0b +
           BMI0 + SBP0 + DBP0 + diabetes0 + CHD0 + stroke0 + headinjury0 +
           depression0 + insomn0,
         data = ukb2)

WM.med1 <- lm(chol0 ~ statin0b + centre2 + age0 + sex + ethnic0b + qualif0b +
                TDI0 + APOE4 + antidep2 + ICV2 + frq_alcohol0 + smoking0 + physact0b +
                BMI0 + SBP0 + DBP0 + diabetes0 + CHD0 + stroke0 + headinjury0 +
                depression0 + insomn0,
              data = ukb2)


WM.chol <- mediate(WM.med1, WM, treat = "statin0b", mediator = "chol0",
                   robustSE = T, sims = 1000)
summary(WM.chol)
```

```
##
## Causal Mediation Analysis
##
## Quasi-Bayesian Confidence Intervals
##
##                  Estimate 95% CI Lower 95% CI Upper p-value
## ACME              204.449      -19.111       458.64   0.074 .
## ADE              -307.248    -1200.456       649.35   0.524
## Total Effect     -102.799    -1037.802       784.52   0.856
## Prop. Mediated     -0.132       -6.093         6.58   0.870
## ---
## Signif. codes:  0 '***' 0.001 '**' 0.01 '*' 0.05 '.' 0.1 ' ' 1
##
## Sample Size Used: 35222
##
```

```
##
## Simulations: 1000
```

Statin, total cholesterol, and peripheral cortical grey matter

```
cortical <- lm(cortical_vol2 ~ statin0b + chol0 + centre2 + age0 + sex + ethnic0b +
                 qualif0b + TDI0 + APOE4 + antidep2 + ICV2 + frq_alcohol0 + smoking0 +
                 physact0b + BMI0 + SBP0 + DBP0 + diabetes0 + CHD0 + stroke0 +
                 headinjury0 + depression0 + insomn0,
          data = ukb2)

cortical.med1 <- lm(chol0 ~ statin0b + centre2 + age0 + sex + ethnic0b +
                 qualif0b + TDI0 + APOE4 + antidep2 + ICV2 + frq_alcohol0 + smoking0 +
                 physact0b + BMI0 + SBP0 + DBP0 + diabetes0 + CHD0 + stroke0 +
                 headinjury0 + depression0 + insomn0,
               data = subset(ukb2, is.na(ukb2$cortical_vol2) == F))


cortical.chol <- mediate(cortical.med1, cortical, treat = "statin0b", mediator = "chol0",
                    robustSE = T, sims = 1000)
summary(cortical.chol)
```

```
##
## Causal Mediation Analysis
##
## Quasi-Bayesian Confidence Intervals
##
##                 Estimate 95% CI Lower 95% CI Upper p-value
## ACME           -1.55e+02    -3.94e+02        94.68   0.204
## ADE            -1.22e+03    -2.14e+03      -318.90   0.012 *
## Total Effect   -1.37e+03    -2.23e+03      -503.32  <2e-16 ***
## Prop. Mediated  1.11e-01    -7.44e-02         0.43   0.204
## ---
## Signif. codes:  0 '***' 0.001 '**' 0.01 '*' 0.05 '.' 0.1 ' ' 1
##
## Sample Size Used: 35222
##
##
## Simulations: 1000
```

Statin, total cholesterol, and WMH

```
WMH <- lm(logWMH_vol2 ~ statin0b + chol0 + centre2 + age0 + sex + ethnic0b + qualif0b +
            TDI0 + APOE4 + antidep2 + ICV2 + frq_alcohol0 + smoking0 + physact0b +
            BMI0 + SBP0 + DBP0 + diabetes0 + CHD0 + stroke0 + headinjury0 +
            depression0 + insomn0,
          data = ukb2)

WMH.med1 <- lm(chol0 ~ statin0b + centre2 + age0 + sex + ethnic0b + qualif0b +
                 TDI0 + APOE4 + antidep2 + ICV2 + frq_alcohol0 + smoking0 + physact0b +
```

```r
                 BMI0 + SBP0 + DBP0 + diabetes0 + CHD0 + stroke0 + headinjury0 +
                 depression0 + insomn0,
                data = subset(ukb2, is.na(ukb2$logWMH_vol2) == F))


WMH.chol <- mediate(WMH.med1, WMH, treat = "statin0b", mediator = "chol0",
                         robustSE = T, sims = 1000)
summary(WMH.chol)
```

```
##
## Causal Mediation Analysis
##
## Quasi-Bayesian Confidence Intervals
##
##                  Estimate 95% CI Lower 95% CI Upper p-value
## ACME              0.00817     -0.00316         0.02    0.17
## ADE               0.10805      0.07135         0.15  <2e-16 ***
## Total Effect      0.11622      0.07912         0.15  <2e-16 ***
## Prop. Mediated   0.07191     -0.02831         0.18    0.17
## ---
## Signif. codes:  0 '***' 0.001 '**' 0.01 '*' 0.05 '.' 0.1 ' ' 1
##
## Sample Size Used: 34102
##
##
## Simulations: 1000
```

SECONDARY ANALYSIS

```r
# Cortical structures with FAST and sub-cortical structures with FIRST
imag <- colnames(ukb2)[c(337:360,362:371,373:380,382:388,390:392,394:396)]
# length(imag)
# 55 imaging variables


# Model 3
mod3 <- multivarlmR2(data = as.data.frame(ukb2),
                    outcome = imag,
                    varexpo = "statin0b",
                    varajust = c("centre2","age0","sex","ethnic0b","qualif0b",
                                 "TDI0","APOE4","antidep2","ICV2",
                                 "frq_alcohol0","smoking0","physact0b",
                                 "BMI0","SBP0","DBP0","diabetes0","CHD0","stroke0",
                                 "headinjury0","depression0","insomn0"),
                    decimal = 2)


mod3 <- as.data.frame(mod3)
mod3$P.adjust <- p.adjust(mod3$P.raw, method = "fdr")
mod3 <- mod3[,c("Outcome","Beta","Lower CI","Upper CI",
                "P value","P.adjust","Adjusted R-squared")]
```

```
mod3
```

```
##                          Outcome    Beta Lower CI Upper CI P value     P.adjust
## 1                    thalam_vol2 -101.53  -142.11   -60.96 <0.0001 3.002218e-05
## 2                      caud_vol2   26.61    -2.77    55.99  0.0758 1.544668e-01
## 3                      puta_vol2   -8.94   -45.36    27.49  0.6306 7.078280e-01
## 4                     palli_vol2  -21.72   -38.72    -4.73  0.0122 4.831096e-02
## 5                      hipp_vol2  -43.25   -76.18   -10.33    0.01 4.721198e-02
## 6                      amyg_vol2  -20.88   -38.21    -3.55  0.0182 5.723677e-02
## 7                     accum_vol2  -17.99   -25.65   -10.34 <0.0001 7.556033e-05
## 8              TemporalPole_vol2 -134.11  -210.78   -57.44   6e-04 5.403626e-03
## 9            SupTemporalAnt_vol2  -34.83    -53.3   -16.36   2e-04 2.413594e-03
## 10          SupTemporalPost_vol2   -6.75   -37.33    23.84  0.6654 7.126323e-01
## 11           MidTemporalAnt_vol2  -42.57   -64.56   -20.58   1e-04 2.042123e-03
## 12          MidTemporalPost_vol2  -14.61   -65.21    35.99  0.5714 6.687145e-01
## 13          MidTemporalOcci_vol2   -21.4   -73.43    30.63  0.4201 5.373579e-01
## 14           InfTemporalAnt_vol2  -24.46   -43.61    -5.31  0.0123 4.831096e-02
## 15          InfTemporalPost_vol2   11.13   -37.16    59.42  0.6515 7.126323e-01
## 16          InfTemporalOcci_vol2   17.53   -24.75     59.8  0.4165 5.373579e-01
## 17       ParahippocampalAnt_vol2  -43.12   -72.19   -14.05  0.0036 2.230255e-02
## 18      ParahippocampalPost_vol2    -8.2   -21.95     5.55  0.2427 3.813378e-01
## 19      TemporalFusiformAnt_vol2  -38.12   -53.45    -22.8 <0.0001 3.002218e-05
## 20     TemporalFusiformPost_vol2   -1.99   -34.08     30.1  0.9032 9.062174e-01
## 21        TempOcciFusiform_vol2   23.79    -8.46    56.04  0.1482 2.629131e-01
## 22            PlanumPolare_vol2   -9.11   -22.56     4.34  0.1842 3.166750e-01
## 23                  Heschl_vol2  -14.11   -28.62      0.4  0.0567 1.299913e-01
## 24         PlanumTemporale_vol2   -11.5   -35.85    12.86  0.3549 5.027525e-01
## 25              SupFrontal_vol2  -23.16  -130.99    84.67  0.6738 7.126323e-01
## 26              MidFrontal_vol2  120.14    15.59    224.7  0.0243 6.685854e-02
## 27         Parsopercularis_vol2  -31.62    -64.5     1.26  0.0594 1.307697e-01
## 28        Parstriangularis_vol2  -13.91   -48.65    20.84  0.4328 5.409831e-01
## 29           FrontalOrbital_vol2  -80.29  -126.65   -33.93   7e-04 5.403626e-03
## 30              Precentral_vol2 -128.24  -235.16   -21.32  0.0187 5.723677e-02
## 31            FrontalMedial_vol2  -10.84   -34.32    12.64  0.3656 5.027525e-01
## 32                  Frontalpole  -60.85  -203.94    82.25  0.4046 5.373579e-01
## 33             Subcallosal_vol2  -23.32   -47.11     0.46  0.0546 1.299913e-01
## 34        FrontalOperculum_vol2  -22.08   -37.98    -6.18  0.0065 3.565007e-02
## 35              Postcentral_vol2  -42.46  -133.93    49.02   0.363 5.027525e-01
## 36       SupParietalLobule_vol2   -49.2  -107.33     8.93  0.0972 1.842594e-01
## 37        SupramarginalAnt_vol2   19.03   -20.45    58.52  0.3447 5.027525e-01
## 38       SupramarginalPost_vol2   18.75   -38.53    76.03  0.5211 6.369409e-01
## 39                 Angular_vol2   39.52   -19.86     98.9  0.1921 3.201451e-01
## 40           Intracalcarine_vol2   -2.63   -46.46    41.19  0.9062 9.062174e-01
## 41               Precuneous_vol2   -54.3  -137.46    28.85  0.2006 3.244327e-01
## 42       ParietalOperculum_vol2   -8.01   -34.74    18.71  0.5567 6.656378e-01
## 43                 Lingual_vol2  -56.44  -107.97    -4.91  0.0318 8.332089e-02
## 44     LateralOccipitalSup_vol2 -199.16  -327.35   -70.97  0.0023 1.600031e-02
## 45     LateralOccipitalInf_vol2    6.79   -66.01     79.6  0.8549 8.871252e-01
## 46                  Cuneal_vol2   -7.65   -36.57    21.26  0.6039 6.919344e-01
## 47       OccipitalFusiform_vol2  -32.98   -69.25     3.29  0.0747 1.544668e-01
## 48           Supracalcarine_vol2   12.43     2.93    21.92  0.0103 4.721198e-02
## 49            OccipitalPole_vol2   -70.2  -156.62    16.23  0.1114 2.042434e-01
## 50           Paracingulate_vol2     -66  -120.18   -11.82   0.017 5.723677e-02
```

```
## 51          CingulateAnt_vol2   68.85    -9.17   146.88  0.0837 1.644066e-01
## 52         CingulatePost_vol2   46.52      2.3    90.73  0.0392 9.804213e-02
## 53               Insular_vol2  -49.93   -90.19    -9.66  0.0151 5.533674e-02
## 54      CentralOpercular_vol2  -39.22   -72.86    -5.59  0.0223 6.446338e-02
## 55 JuxtapositionalLobule_vol2  -21.51   -62.45    19.43  0.3032 4.632047e-01
##    Adjusted R-squared
## 1              0.6027
## 2              0.3546
## 3              0.4701
## 4              0.2786
## 5              0.2644
## 6              0.1601
## 7              0.2948
## 8              0.4246
## 9              0.3069
## 10             0.3715
## 11             0.3222
## 12             0.4344
## 13             0.3014
## 14             0.2293
## 15             0.3293
## 16             0.3082
## 17             0.3696
## 18             0.2448
## 19             0.3331
## 20             0.4247
## 21             0.3859
## 22             0.4282
## 23             0.3942
## 24             0.3868
## 25             0.3579
## 26             0.4171
## 27             0.2434
## 28             0.1957
## 29             0.5038
## 30             0.4319
## 31             0.2218
## 32              0.654
## 33             0.5011
## 34             0.3029
## 35             0.3829
## 36             0.2362
## 37              0.241
## 38             0.2833
## 39             0.2632
## 40             0.2506
## 41             0.5358
## 42             0.3769
## 43             0.5295
## 44             0.4892
## 45             0.4049
## 46             0.3091
## 47             0.3811
## 48             0.2981
```

```
## 49              0.3833
## 50              0.4144
## 51              0.2691
## 52              0.5487
## 53              0.5092
## 54              0.4996
## 55                0.21
```

SENSITIVITY ANALYSIS

Never users versus long-term statin users

```
## Create a variable for long-term statin users

ukb2$statin_t02 <- rep(NA, dim(ukb2)[1])
# 0: never used statin
ukb2$statin_t02[ukb2$statin0b == 0 & ukb2$statin2b == 0] <- 0
# 1: started using statin at the imaging visit (removed)
ukb2$statin_t02[ukb2$statin0b == 0 & ukb2$statin2b == 1] <- 1
# 2: reported using statin at recruitment but not at the imaging visit (removed)
ukb2$statin_t02[ukb2$statin0b == 1 & ukb2$statin2b == 0] <- 2
# 3: continuously used statin between recruitment and imaging visit
ukb2$statin_t02[ukb2$statin0b == 1 & ukb2$statin2b == 1] <- 3

ukb2$statin_t02 <- as.factor(ukb2$statin_t02)
table(ukb2$statin_t02)
```

```
##
##     0     1     2     3
## 31400  4817   828  2457
```

```
ukb3 <- ukb2 %>% filter(ukb2$statin_t02 == "0" | ukb2$statin_t02 == "3")
table(ukb3$statin0b)
```

```
##
##     0     1
## 31400  2457
```

```
# Model 1
m.longt1 <- multivarlmR2(data = as.data.frame(ukb3),
                         outcome = imagsel,
                         varexpo = "statin0b",
                         varajust = c("centre2","age0","sex","ethnic0b","qualif0b",
                                      "TDI0","APOE4","antidep2","ICV2"),
                         decimal = 2)

m.longt1 <- as.data.frame(m.longt1)
m.longt1$Model <- rep("I",4)

# Model 2
m.longt2 <- multivarlmR2(data = as.data.frame(ukb3),
```

```r
                         outcome = imagsel,
                         varexpo = "statin0b",
                         varajust = c("centre2","age0","sex","ethnic0b","qualif0b",
                                      "TDI0","APOE4","antidep2","ICV2",
                                      "frq_alcohol0","smoking0","physact0b"),
                         decimal = 2)

m.longt2 <- as.data.frame(m.longt2)
m.longt2$Model <- rep("II",4)

# Model 3
m.longt3 <- multivarlmR2(data = as.data.frame(ukb3),
                         outcome = imagsel,
                         varexpo = "statin0b",
                         varajust = c("centre2","age0","sex","ethnic0b","qualif0b",
                                      "TDI0","APOE4","antidep2","ICV2",
                                      "frq_alcohol0","smoking0","physact0b","BMI0",
                                      "SBP0","DBP0","diabetes0","CHD0","stroke0",
                                      "headinjury0","depression0","insomn0"),
                         decimal = 2)

m.longt3 <- as.data.frame(m.longt3)
m.longt3$Model <- rep("III",4)

m.longt <- rbind(m.longt1, m.longt2, m.longt3)
m.longt <- m.longt %>% arrange(Outcome)
m.longt <- m.longt[,-8]

m.longt
```

```
##           Outcome   Exposure     Beta     SE Lower CI Upper CI P value
## 1        GM_vol2 statin0b_1 -3647.08 395.56 -4422.39 -2871.78 <0.0001
## 2        GM_vol2 statin0b_1 -3455.04 399.91 -4238.88 -2671.19 <0.0001
## 3        GM_vol2 statin0b_1 -1795.79 457.85 -2693.19  -898.39 <0.0001
## 4        WM_vol2 statin0b_1  1173.29 393.63   401.75  1944.83  0.0029
## 5        WM_vol2 statin0b_1   973.78 399.32   191.11  1756.46  0.0147
## 6        WM_vol2 statin0b_1    60.23 458.05  -837.56   958.02  0.8954
## 7   cortical_vol2 statin0b_1 -2374.54 393.39 -3145.59 -1603.49 <0.0001
## 8   cortical_vol2 statin0b_1 -2234.11 398.02 -3014.24 -1453.97 <0.0001
## 9   cortical_vol2 statin0b_1 -1379.86 457.13 -2275.84  -483.87  0.0025
## 10    logWMH_vol2 statin0b_1     0.17   0.02     0.13     0.21 <0.0001
## 11    logWMH_vol2 statin0b_1     0.16   0.02     0.12      0.2 <0.0001
## 12    logWMH_vol2 statin0b_1      0.1   0.02     0.06     0.14 <0.0001
##    Adjusted R-squared Model
## 1               0.896     I
## 2              0.8969    II
## 3              0.8977   III
## 4              0.9158     I
## 5               0.916    II
## 6              0.9163   III
## 7              0.8508     I
## 8              0.8518    II
## 9              0.8521   III
```

```
## 10             0.2749     I
## 11             0.2773    II
## 12             0.2956   III
```

Replace self-reported medical history variables with variables based on ICD10 diagnoses

```
mod3ICD <- multivarlmR2(data = as.data.frame(ukb2),
                        outcome = imagsel,
                        varexpo = "statin0b",
                        varajust = c("centre2","age0","sex","ethnic0b","qualif0b",
                                     "TDI0","APOE4","antidep2","ICV2",
                                     "frq_alcohol0","smoking0","physact0b","BMI0",
                                     "SBP0","DBP0","diabetesICD0","CHD_ICD0",
                                     "stroke_ICD0","headinjuryICD0","depressionICD0"),
                    decimal = 2)

mod3ICD <- as.data.frame(mod3ICD)
mod3ICD$Model <- rep("III",4)
mod3ICD <- mod3ICD[,-8]

mod3ICD
```

```
##         Outcome   Exposure     Beta     SE Lower CI Upper CI P value
## 1       GM_vol2 statin0b_1 -2596.81 380.35 -3342.31 -1851.32 <0.0001
## 2       WM_vol2 statin0b_1   222.54 380.17  -522.61   967.69  0.5583
## 3 cortical_vol2 statin0b_1 -2303.73 378.14 -3044.89 -1562.58 <0.0001
## 4   logWMH_vol2 statin0b_1     0.14   0.02     0.11     0.18 <0.0001
##   Adjusted R-squared Model
## 1              0.894   III
## 2             0.9135   III
## 3             0.8475   III
## 4             0.3001   III
```
